# Distinct molecular mechanisms underlying clinically relevant subtypes of breast cancer: gene expression analyses across three different platforms

**DOI:** 10.1186/1471-2164-7-127

**Published:** 2006-05-26

**Authors:** Therese Sørlie, Yulei Wang, Chunlin Xiao, Hilde Johnsen, Bjørn Naume, Raymond R Samaha, Anne-Lise Børresen-Dale

**Affiliations:** 1 Department of Genetics, Institute for Cancer Research, Rikshospitalet-Radiumhospitalet Medical Center, N-0310 Oslo, Norway; 2 Applied Biosystems, Foster City, CA 94404, USA; 3 Celera Genomics, Rockville, MD 20850, USA; 4 Department of Oncology, Rikshospitalet-Radiumhospitalet Medical Center, N-0310 Oslo, Norway; 5 Medical Faculty, University of Oslo, N-0310 Oslo, Norway

## Abstract

**Background:**

Gene expression profiling has been used to define molecular phenotypes of complex diseases such as breast cancer. The luminal A and basal-like subtypes have been repeatedly identified and validated as the two main subtypes out of a total of five molecular subtypes of breast cancer. These two are associated with distinctly different gene expression patterns and more importantly, a significant difference in clinical outcome. To further validate and more thoroughly characterize these two subtypes at the molecular level in tumors at an early stage, we report a gene expression profiling study using three different DNA microarray platforms.

**Results:**

Expression data from 20 tumor biopsies of early stage breast carcinomas were generated on three different DNA microarray platforms; Applied Biosystems Human Genome Survey Microarrays, Stanford cDNA Microarrays and Agilent's Whole Human Genome Oligo Microarrays, and the resulting gene expression patterns were analyzed. Both unsupervised and supervised analyses identified the different clinically relevant subtypes of breast tumours, and the results were consistent across all three platforms. Gene classification and biological pathway analyses of the genes differentially expressed between the two main subtypes revealed different molecular mechanisms descriptive of the two expression-based subtypes: Signature genes of the luminal A subtype were over-represented by genes involved in fatty acid metabolism and steroid hormone-mediated signaling pathways, in particular estrogen receptor signaling, while signature genes of the basal-like subtype were over-represented by genes involved in cell proliferation and differentiation, p21-mediated pathway, and G1-S checkpoint of cell cycle-signaling pathways. A minimal set of 54 genes that best discriminated the two subtypes was identified using the combined data sets generated from the three different array platforms. These predictor genes were further verified by TaqMan^® ^Gene Expression assays.

**Conclusion:**

We have identified and validated the two main previously defined clinically relevant subtypes, luminal A and basal-like, in a small set of early stage breast carcinomas. Signature genes characterizing these two subtypes revealed that distinct molecular mechanisms might have been pre-programmed at an early stage in different subtypes of the disease. Our results provide further evidence that these breast tumor subtypes represent biologically distinct disease entities and may require different therapeutic strategies. Finally, validated by multiple gene expression platforms, including quantitative PCR, the set of 54 predictor genes identified in this study may define potential prognostic molecular markers for breast cancer.

## Background

Breast cancer is a complex disease and although recent research has emphasized the heterogeneity of the disease, much of its biology remains poorly understood. In particular, genomic tools such as DNA microarrays hold great potential for the deciphering of the molecular patterns of tumors and the identification of new and improved clinical markers. Gene expression profiling has been used extensively over the last few years to analyze breast tumors and has resulted in several gene signatures associated with different clinical parameters [1-7]. Using an unsupervised approach, we have identified five clinically relevant subtypes of breast tumors [4,8], which have been further validated in independent data sets [3,9-14]. Of these, the two main subtypes are associated with the most significant difference in clinical outcome: Patients with luminal A type tumors are facing a relatively good prognosis, whereas patients with basal-like tumors experience a much shorter overall-and disease-free survival period [10]. They are also associated with differences in pathologic response to chemotherapy [9,10,15].

Our earlier findings suggested that the distinct expression patterns of the tumor subtypes and the significant differences in disease outcome are likely to be caused, at least in part, by alterations in specific cellular pathways and/or different cell type origin. The luminal A type tumors are characterized by high expression of the estrogen receptor (*ESR1*) and a handful of other genes generally co-expressed with *ESR1*, many of which are genes typically expressed in the luminal epithelium that lines the ducts. The basal-like tumors on the other hand, are characterized by high expression of some basal epithelial markers such as *KRT5*, *KRT17 *and *LAMC2 *(laminin), and many cell cycle-regulated genes [16,17]. A more thorough characterization of the molecular basis underlying these subtypes, in particular in breast carcinomas at an earlier stage, will help us to better understand breast cancer diseases at cellular levels and hopefully provide new molecular prognostic markers and targets for therapy.

In this study, we profiled 20 samples from early breast carcinomas (T1/T2) using three different microarray platforms to address the question of uniformity of the breast cancer phenotypes with different technologies. We identified differentially expressed genes between the two main tumor subtypes, luminal A and basal-like, and subjected these to protein classification and pathway analyses. Finally, we identified a minimum set of genes with the best possible predictive power for an expression-based prognostic assay for the two clinically relevant subtypes of breast cancer.

## Results

### Identification and validation of tumor subtypes in early breast cancer

**Figure 1:**
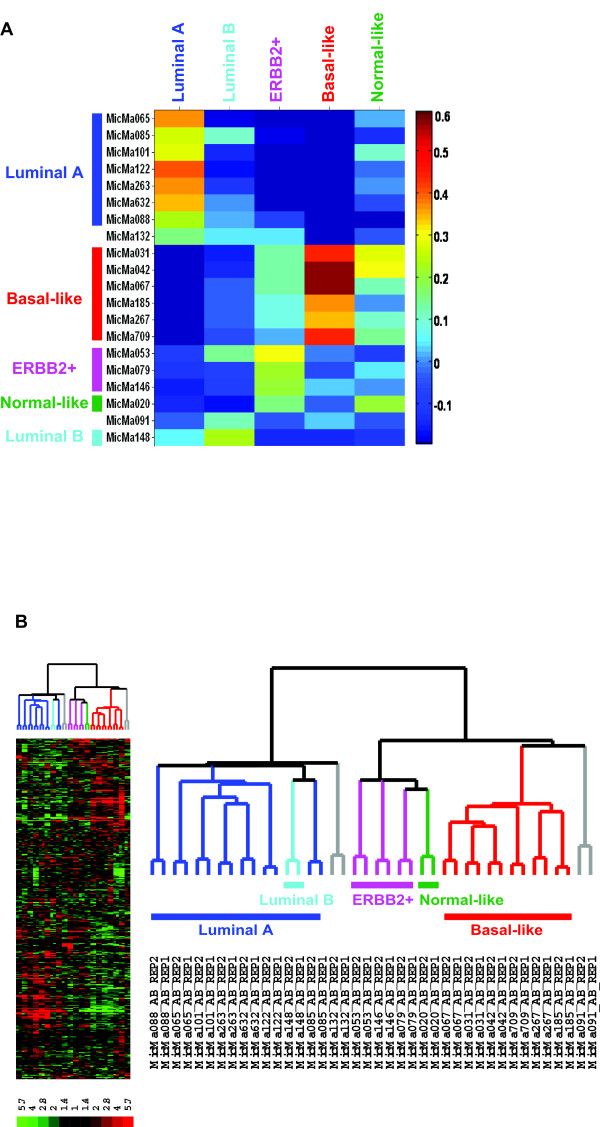
**Unsupervised approach to identify breast cancer subtypes**. (A) Correlation of breast tumor samples with the previously identified five subtypes of breast tumors. 526 out of 552 previously identified "intrinsic" genes were cross-mapped to Applied Biosystems Human Genome Survey Microarray and used for centroid correlation analysis and hierarchical clustering. Correlations with the centroids of the five subtypes were calculated for each sample from this study (the two microarray replicates for each sample were averaged). Samples were assigned to a subtype with which it showed the highest correlation using a cutoff value of 0.2. (B) Unsupervised hierarchical clustering of 20 breast tumor tissues analyzed by AB arrays using the 526 mapped intrinsic genes (the two microarray replicates for each sample are shown). The level of expression of each gene in each sample, relative to the median level of expression of that gene across all the samples, is represented using a red-black-green color scale as shown in the key (green: below median; black: equal to median; red: above median). (Left panel): Scaled down representation of the entire cluster of the 526 intrinsic genes and 20 tissue samples. (Right panel): Experimental dendrogram displaying the clustering of the tumors into three distinct subgroups. Branches are color-coded according to the subtype with which the corresponding tumor sample showed the highest correlation. Tumors with low correlation (< 0.2) with a specific subtype are indicated by gray branches.

Our first approach for identifying the previously described tumor subtypes was to correlate the expression patterns of the 20 early stage breast carcinomas analyzed by Applied Biosystems Expression Array System with the previously published expression centroids of the five tumors subtypes [10]. The correlation matrix summarized as a heat map is shown in Figure 1A. The previously identified subtypes were evident also in this small tumor set. Using a correlation coefficient cutoff of 0.2, six tumors were defined as basal-like, seven were luminal A, three tumors were ERBB2+, one luminal B and finally, one was identified as normal breast-like. Two tumors remained unclassified using the 0.2 threshold.

Unsupervised hierarchical clustering of the 20 tumors using the 526 mapped intrinsic genes identified three sub-clusters of samples based on their expression patterns (Figure 1B). Individual dendrogram branches are colored according to the strongest correlation of the corresponding tumor with a subtype centroid. Among these sub-groups, the clearest distinction was observed between the luminal A (ER+) and the basal-like (ER-) subtypes, as has been repeatedly reported.

**Figure 2:**
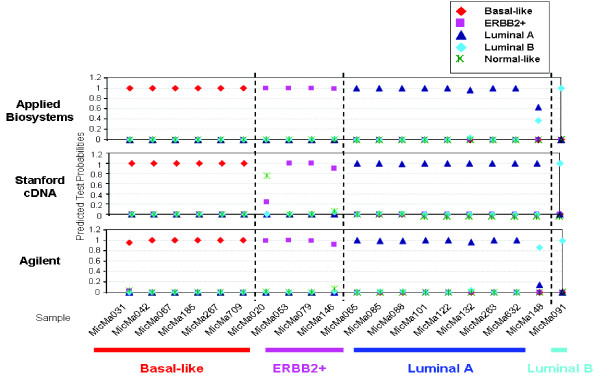
**Prediction of tumor subtype by Prediction Analysis of Microarrays (PAM)**. 428 genes were selected at a threshold of 1.0 that separated the two subtypes with the lowest overall misclassification rate of 5% (data not shown). Predicted probabilities of subtype for each tumor sample analyzed on Applied Biosystems Human Genome Survey Microarrays (top panel), Stanford Human cDNA arrays (middle panel) and Agilent Whole Human Genome Microarrays (bottom panel) were computed using the predictive model built using these 428 predictor genes.

As a second approach to validate the tumor subtypes in this data set, we applied a supervised analysis using "Nearest Shrunken Centroid Classifier" and the PAM software. We took the previously published 122 Norway/Stanford data set [10] and the mapped 526 genes as the training set to identify predictor genes for the five subtypes. With a threshold (Δ) of 1 and 10-fold cross validation, we built a classifier containing 428 genes which gave < 5% misclassification error (data not shown). We then used this classifier to predict the subtypes of the 20 tumors analyzed in this study. The prediction results from the supervised analysis were overall consistent with the unsupervised analysis using hierarchical clustering and centroid correlation analysis: 6 tumors were predicted to be basal-like, 8 tumors were predicted to be luminal A, 4 were determined as ERBB2+, 1 as luminal B, and 1 was unclassifiable (Figure 2, top panel). In other words, all but one sample were assigned to a subtype with high prediction probability; however, it is worth to note that the prediction accuracy may be over-estimated as the predictor genes are a subset of those used to define the subtypes in the first place.

### Confirmation of the luminal A and basal-like subtypes using different array platforms

**Figure 3:**
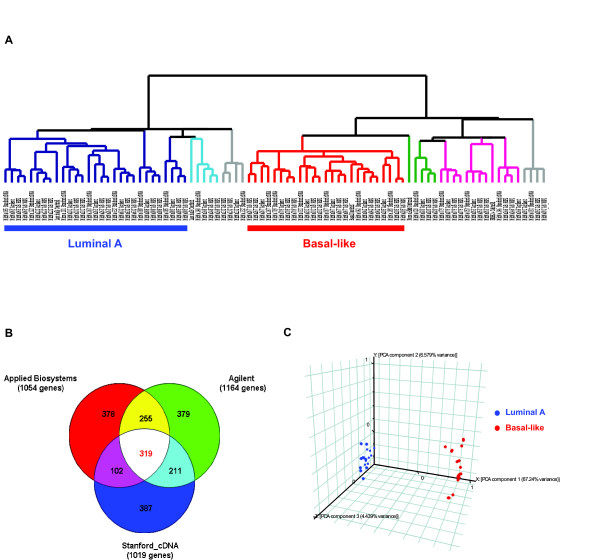
**Validation of the luminal A and basal-like subtypes using three microarray platforms**. (A) Unsupervised hierarchical clustering of 20 breast tumor tissues analyzed by Applied Biosystems (two replicates per sample), Stanford cDNA and Agilent arrays using the 510 mapped intrinsic genes. The data from each platform were first transformed independently and then combined for clustering: the level of expression of each gene in each sample (Applied Biosystems microarrays: normalized signal intensity; Stanford cDNA and Agilent microarrays: normalized log2 ratio of the sample vs. the reference (UHR)) was transformed into a log2 ratio relative to the median level of expression of that gene across all the samples within the data set of the given platform. The experimental dendrogram displays the clustering of the tumors into distinct subgroups. Branches are color-coded according to the subtype with which the corresponding tumor sample showed the highest correlation. Tumors with low correlation (< 0.2) with a specific subtype are indicated by gray branches. Luminal A subtype (dark blue) and basal-like subtype (red). (B) Venn Diagram of the most differentially expressed genes identified by all three different array platforms; 319 genes were identified as the common signature genes using ANOVA analysis and the following criteria: (1) > 2-fold change between the two subtypes, and (2) False discovery rate < 5%. (C) PCA analysis of luminal A and basal-like samples using a minimum set of 54 genes identified by PAM analysis. Data sets generated from three array platforms (48 arrays total, two replicates per sample in the Applied Biosystems data set) on 6 luminal A and 6 basal-like tumor samples using the 319 common signature genes were used as training set for the PAM analysis.

We also analyzed the same 20 tumor samples on Stanford Human cDNA microarrays and Agilent Whole Human Genome Oligo Microarrays. Centroid correlation analysis was performed as described above using the 510 common intrinsic genes mapped among all three platforms. Unsupervised hierarchical clustering of the three data sets revealed the exact same subgroups of tumors, with the luminal A and the basal-like subtypes as the most predominant and distinct (Figure 3A). PAM analysis showed consistent prediction of subtype for each tumor sample from the three data sets except for two samples (MicMa148 and MicMa 020; see Figure 2). Overall, there was consistency in identifying these biological subtypes between all three platforms.

### Molecular characterization of luminal A and basal-like subtypes of breast tumors

**Figure 4:**
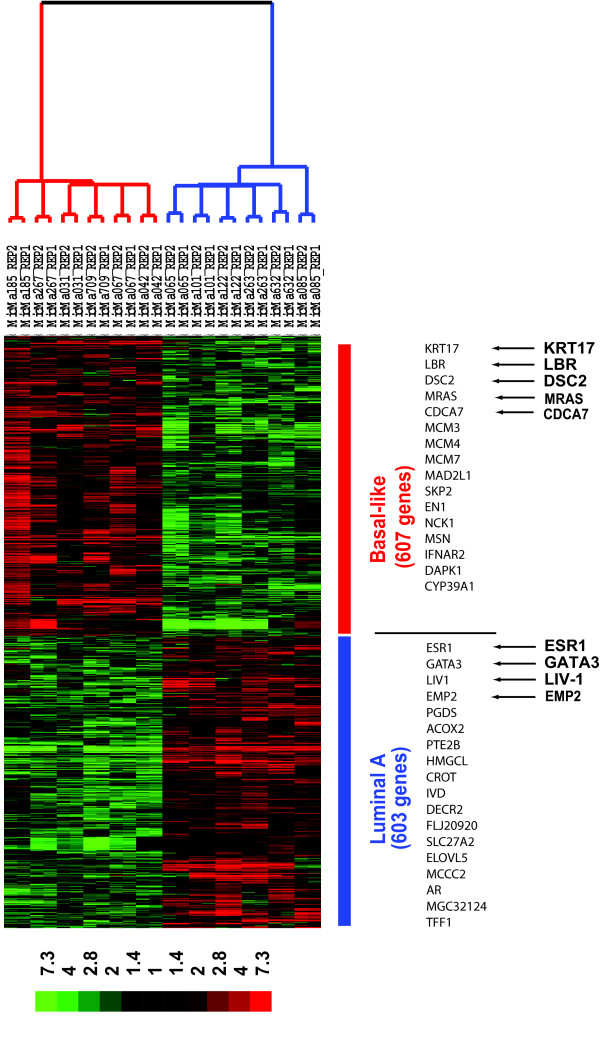
**Two-dimensional cluster diagram of the 1210 signature genes characterizing the luminal A and basal-like subtypes**. ANOVA analysis was performed on 6 luminal A samples and 6 basal-like samples, coupled with Benjamini and Hochberg False Discovery Rate multiple testing corrections. The two subtypes of breast tumors (two replicates per sample) were clustered into distinct clusters with reversed gene expression patterns: highly expressed in luminal A (bottom, 613 probes representing 603 genes) and highly expressed in basal (top, 631 probes representing 607 genes). The color scheme of the heat map was described in legend to Figure 1B and is shown in the key. Branches in the dendrogram are color-coded according to the subtypes: blue, luminal A; red, basal-like.

To molecularly characterize these two subtypes, we first identified the most differentially expressed genes as the "signature" genes using data from the Applied Biosystems Expression Arrays, as this system provides a comprehensive coverage of the genome including genes not covered by other commercial microarrays. ANOVA analysis coupled with Benjamini and Hochberg False Discovery Rate multiple testing corrections were performed on six luminal A samples and six basal-like tumor samples (to be most stringent in this analysis, samples with centroid correlation coefficient > 0.3 were used, therefore, MicMa088 and MicMa 132 were excluded, see Figure 1A). 1210 genes represented by 1244 probes were identified as the "signature" genes meeting the following criteria: (1) Detectable (signal to noise > 3) in > 50% samples; (2) > 2-fold change between the two subtypes, and (3) False discovery rate < 5% (see Additional file 1). Figure 4 displays a hierarchical clustering diagram of the 12 tumor samples using these 1210 signature genes. Among the signature genes, 613 probes (603 genes) were specifically over-expressed in luminal A type tumors (luminal A "signature" genes), which included some previously identified markers, such as *ESR1*, *GATA3 *and *LIV1 *[10], as well as many other potential marker genes for this subtype. One example is the *EMP2 *gene, which encodes a tetra-span membrane protein that has been reported to suppress B-cell lymphoma tumorigenicity [18]. In the basal-like tumors, 631 probes (607 genes) were specifically over-expressed (basal-like "signature" genes), and these included markers such as *KRT17*, the Lamin B receptor (*LBR*) and *DSC2*. Two interesting genes specifically overexpressed in these tumors were *MRAS*, a well known oncoprotein of the RAS superfamily whose mutant forms may transform mammary epithelial cells [19], and *CDCA7*, a direct target of the *MYC *oncogene [20].

To depict more detailed molecular portraits of these two subtypes, we analyzed which biological processes were over-represented by these signature genes using the PANTHER™ Protein Classification System analysis. The most significantly over-represented biological processes are listed in Table 1 (upper panel). Not surprisingly, very different processes are underlined by the signature genes of the two subtypes: For luminal A, the most over-represented biological processes (*p *< 0.001) include fatty acid metabolism (e.g. *PGDS*, *ACOX2*, *PTE2B*, *HMGCL*, *CROT*, *IVD*, *DECR2*, *FLJ20920*, SLC27A2, *ELOVL5*, and *MCCC2*) and steroid hormone mediated signaling (e.g. *CRABP2*, *AR*, *MGC32124*, *ESR1*), whereas for the basal-like subtype, the most over-enriched processes (*p *< 0.001, only top five listed) include ones that involve many cancer "hallmark" genes, such as the cell cycle, cell proliferation and differentiation, protein phosphorylation, B-cell-and antibody-mediated immunity.

**Table 1:** Annotation and biological pathway analysis.

**Protein Classifications and Pathways**	**Number of Overlapping Genes**	**Random Overlapping p value**
**PANTHER™ Protein Classification System (*p *< 0.001)**		
***Luminal A subtype***		
Fatty acid metabolism	11	1.22E-04
Steroid hormone-mediated signaling	5	8.09E-04
		
***Basal-like subtype***		
Cell cycle	55	2.50E-13
Cell proliferation and differentiation	40	9.62E-08
Protein phosphorylation	34	2.31E-07
B-cell-and antibody-mediated immunity	17	2.63E-07
Cell cycle control	25	2.38E-06
		
**Jubilant PathArt™ Pathways (*p *< 0.01)**		
***Luminal A subtype***		
ER Signaling Pathway	6	2.80E-04
Retinoic Acid Signaling Pathway	4	4.46E-04
Nucleotide Excision Repair Pathway	4	1.92E-03
IL6 Signaling Pathway	6	4.73E-03
EGF Signaling Pathway	7	4.92E-03
		
***Basal-like subtype***		
p21 Mediated Pathway	15	9.14E-13
G1-S Checkpoint Pathway	10	1.03E-08
FAS Mediated Pathway	3	7.49E-04
p53 Signaling Pathway	7	8.45E-04

Upper part: The most significant biological processes (PANTHER™ Protein Classification System) over-represented by the luminal A subtype and the basal-like subtype (p < 0.001, top 5 are shown); Lower part: The most significant cellular pathways (PathArt™) over-represented by the luminal A subtype and the basal-like subtype (p < 0.01).

In a similar fashion, we also analyzed which cellular pathways played critical roles for defining the two distinct subtypes using Jubilant's PathArt™ database. Table 1 (lower panel) shows the top five PathArt™ pathways over-represented (*p *< 0.01) by the genes characteristic for luminal A and the basal-like subtypes, respectively. Again, quite distinct pathways were found to underlie the two subtypes of breast tumors. As expected, the most over-represented pathway activated in the luminal A subtype is the ER signaling pathway (see Additional file 2A): 6 genes within the ER signaling pathway were significantly up-regulated in these tumors, including the estrogen receptor 1 (*ESR1*) and the estrogen-induced gene trefoil factor 1 (*TFF1*). This is consistent with the previous findings that the luminal A type tumors over-express *ESR1 *and other estrogen-responsive genes and therefore are responsive to adjuvant hormonal treatment [10,21]. On the other hand, the biological pathways underlying the basal-like subtype are well known cancer-associated pathways. For example, fifteen genes in the p21 (*CDKN1A*) pathway were coordinately over-expressed in the basal-like tumors, many of these, such as *MCM3*, *MCM4*, *MCM7 *and *MAD2L1 *play critical roles in cell proliferation and DNA replication (see Additional file 2B). Among many genes in this pathway, *SKP2 *was found over-expressed in basal-like tumors; it encodes a protein involved in the degradation of another cyclin-dependent kinase inhibitor p27 (*CDKN1B*) and recently reported to be over-expressed in many tumor types and to correlate with poor prognosis [22,23].

Our findings confirm the existence of the intrinsic tumor subtypes in these early breast cancer specimens, as has been reported by others [3,13] and indicate that the cellular processes revealed by gene expression profiling have been programmed at earlier stages of tumorigenesis.

### Identification of the best set of prognostic genes discriminating the luminal A and basal-like subtypes of breast tumors

In an effort to identify a minimal set of genes that best characterize the two subtypes and can form the basis for a prognostic gene profile, we performed ANOVA analysis on the data sets generated on each of the three array platforms using the 16,611 common genes (see Additional file 3) and the same six luminal A and six basal-like tumor samples. Differentially expressed genes between the two subtypes were determined for each platform using the same criteria: (1) > 2-fold change between the two subtypes, and (2) False discovery rate < 5%. From these, 319 genes were identified as the common signature genes (Figure 3B). The combined data sets generated from the three platforms using expression data from these 319 common genes was then used as the training set to perform PAM analysis for the identification of the minimal set of genes to best discriminate luminal A and basal-like tumors. Ten-fold cross validation and a threshold of Δ = 1.9 identified 54 genes with a misclassification error of ~ 16.7%. The genes are listed in Additional file 4. Principle Component Analysis on the 12 tumor samples profiled on all three array platforms using these 54 genes clearly separated the two subtypes of breast tumors (Figure 3C).

### Real-time PCR validation using TaqMan^® ^Gene Expression Assays

**Figure 5:**
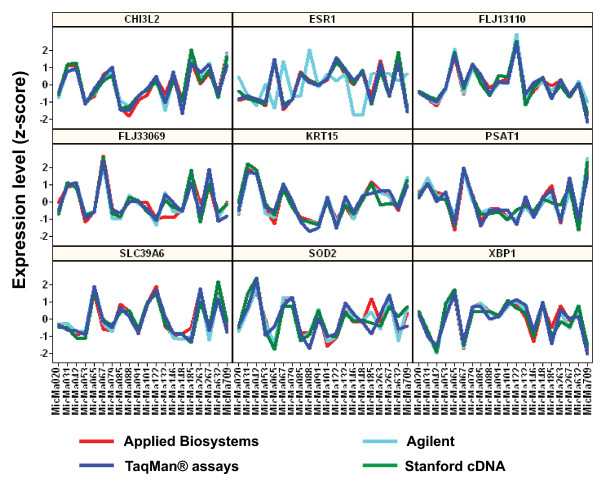
**Validation of potential prognostic markers by Taqman^® ^assay-based real-time PCR**. 85 marker genes including the minimal set of 54 genes identified to best distinguish the luminal A and the basal-like subtypes were validated by TaqMan^® ^Gene Expression assays. Profile correlation analysis showed the expression profile across the 20 tumor samples determined by the three microarray platforms and by TaqMan^® ^Gene Expression assays are highly correlated with median correlation coefficient R > 0.9 and a rate of good correlation (R > 0.8) of 81–88%.

**Figure 6:**
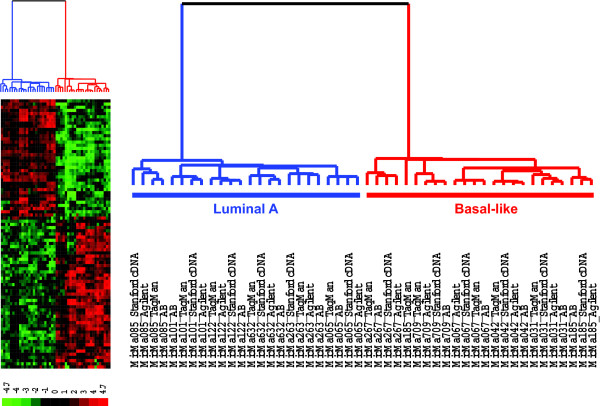
**Hierarchical clustering analysis of expression data from the TaqMan^® ^assays and the three microarray platforms across the 85 marker genes**. The same tumor sample analyzed by the three microarray platforms and the TaqMan^® ^Gene Expression assays were clustered together except for one sample (MicMa185). For the Applied Biosystems microarrays, the mean expression values of the two microarray replicates were presented. The transformed z-scores were represented using a red-black-green color scale as shown in the key (green: below mean; black: equal to mean; red: above mean).

To further validate these prognostic markers, we selected altogether 85 genes (the 54 minimal set of predictor genes plus an additional 14 top-ranking predictor genes from the PAM analysis, 10 previously identified markers (from the intrinsic gene list) and 7 selected G-protein coupled receptors (GPCRs) and secreted proteins) and performed real-time PCR using TaqMan^® ^Gene Expression Assays (see Additional file 5). Profile correlation analysis showed that the expression profiles across the 20 tumor samples determined by each array platform and by TaqMan^® ^assays are highly correlated (Figure 5) (median correlation coefficient R > 0.9, the rate of good correlation (Pearson correlation coefficient, R > 0.8) varied from 81–88% for the three array platforms (Table 2). Hierarchical clustering analysis not only demonstrated excellent separation of the luminal A and basal-like subtypes, but that the same tumor sample analyzed by three microarray platforms and the TaqMan^® ^assays were clustered together by tumor sample rather than by method (Figure 6).

**Table 2:** Pearson correlation between gene expression profiles determined by TaqMan real-time PCR and three DNA microarray platforms

Pearson Correction with TaqMan Assay	Applied Biosystems	Stanford cDNA Array	Agilent
Good (cor.coeff. > 0.8)	75 (88%)	69 (81%)	73 (86%)
Consistent (0 < cor.coeff. < 0.8)	8 (9%)	15 (18%)	9 (11%)
Anti-Correlation (cor.coeff. < 0)	2 (2%)	1(1%)	3 (4%)
Median	0.91	0.90	0.91

## Discussion

Analyses of gene expression patterns from thousands of genes using DNA microarrays have demonstrated great diversity among tumors arising in the same organ and with apparently similar histopathology. This has raised hopes that classification schemes based on molecular profiling may better capture the complex behavior of tumors and lead to improved prognostication and tailor-made therapeutic strategies. We were the first to identify that specific subclasses of breast cancer, based on gene expression profiling, were distinct biological entities and associated with significant differences in outcome for patients with locally advanced breast cancer [4]. Subsequently, this has been validated both by us and other groups in different types of breast cancer patient cohorts [3,9-13]. Here, we could confirm the existence of the molecular subtypes of breast tumors also in early breast cancer (T1/T2) using three different microarray platforms. Due to the small sample size reported here, only the luminal A and basal-like groups could be robustly identified, although the other less represented subtypes could also be recognized. These two subtypes are easily distinguishable in several tumor data sets and their expression profiles seem to be anti-correlated, as also has been shown for breast cancer cell lines [24], but the cellular pathways affected are not known in detail. We show here that the differences in gene expression patterns between the two main subtypes reflect levels of activation of distinct signaling pathways. These changes might have been pre-programmed already at a relatively early stage in the progression of the cancer and hence, imply that the fate of the tumor is already set. This is in accordance with previous reports on breast cancer [3,9,10,25-27]. Other groups have analyzed gene expression in DCIS (ductal carcinoma in situ) for comparison with invasive carcinomas and highlighted transcripts that may be important for transformation and invasion [13,28,29]. Extensive studies of DCIS and other pre-invasive stages of tumors will further enlighten this hypothesis and substantiate the value of gene expression-based classification in prognosis of breast cancer at an early stage.

Specifically in this study, a more in-depth molecular characterization of these phenotypes of breast cancer was carried out and provided new insights into the biology of the disease at the molecular level. The distinct and characteristic molecular mechanisms revealed by the protein classification and biological pathway analysis, provided further evidence that these molecular subtypes represent biologically distinct disease entities and may require different therapeutic strategies. For example, our results indicated that the luminal A subtype showed coordinated activation of genes involved in steroid/estrogen signaling and fatty acid metabolism. Fatty acid synthase (FAS)-dependent endogenous fatty acid synthetic activity has been found to be abnormally elevated in a subset of aggressive breast carcinomas [30], in particular ERBB2-overexpressing tumors [31], whereas here, high expression of many genes involved in fatty acid/lipid metabolism and degradation were coupled to the luminal A phenotype, know to be associated with a relatively good prognosis [10]. Although no correlation between fatty acid metabolism and estrogen and progesterone receptor expression status of tumors has been documented in cancer, our results may indicate some level of cross-talk between fatty acid metabolism and steroid signaling that may have effects on apoptosis and cell proliferation and possibly hormonal treatment in this subtype of breast cancer. Indeed, it has been speculated that some lipids may modulate steroid metabolism [32].

Such molecular profiling of clinically relevant subtypes of breast tumors provide opportunities for identification of novel targets that can be exploited for targeted therapeutics of the disease. Among the 1210 genes most differentially expressed between luminal A and basal-like tumors, 145 are secreted proteins based on the prediction methodology published in a recent paper [33]. A variety of biomolecules are secreted proteins such as cytokines, chemokines, hormones and digestive enzymes that play pivotal biological regulatory roles and are very important sources for protein therapeutics. We also identified five G protein-coupled receptors (GPCRs) among these signature genes, a gene family well established as small molecule drug targets.

Although we analyzed only 20 tumor biopsies, data were collected using three different microarray platforms; a two-color fluorescent-based cDNA microarray, a 60-mer oligo microarray using two-color fluorescence detection and a 60-mer oligo microarray using chemiluminescence detection. Of 16,611 common genes among these three platforms, 1019, 1054 and 1164 genes, respectively, were identified to be differentially expressed between luminal A and basal-like tumors. Of these, 319 genes were common to all three technologies, which correspond to an overall consistency of 30%. These numbers could prove to be even higher if a more accurate probe match by sequence rather than gene identifiers would be performed, as has recently been shown [34]. A few studies have recently been published that aimed to compare variability and consistency between microarray platforms and with different results [35-37]. Our study shows that although there is variability between the platforms, the gene expression profiles emerging from using all three technologies are highly correlated to the biological variation in the data and the same tumor subtype pattern was identified with all three methods.

The minimal set of 54 genes that best characterized luminal A and basal-like subtypes was identified based on differential expressed genes on all three platforms and validated by using TaqMan^® ^assays. Convincingly, clustering of expression data from all four methods grouped the experiments together by tumor sample of origin and not by platform. Hence, these genes provide a robust set of potential prognostic molecular markers, but which covers only the two main subtypes. More thorough characterization on significantly larger sample sizes is needed to provide prognostic predictor sets for all subtypes.

## Conclusion

We have validated and characterized the two main previously defined clinically relevant subtypes, luminal A and basal-like, in early stage breast carcinomas, using three different DNA microarray platforms. Signature gene profiles characterizing these two subtypes revealed that distinct molecular mechanisms might have been pre-programmed at an earlier stage in different subtypes of the disease. Our results provide further evidence that these breast tumor subtypes represent biologically distinct disease entities and may require different therapeutic strategies. Finally, validated by the gene expression platforms and TaqMan^® ^assay-based real time PCR, the set of 54 predictor genes identified in this study defines a set of highly-validated and potential prognostic molecular markers for these subtypes of breast cancer.

## Methods

### Tumor samples and RNA preparation

Tissue samples from a pilot set of 20 breast carcinomas, a small subset of a larger series of 920 unselected early-stage breast cancer patients, for which informed, written consent was obtained by the Regional Ethical Committee [38], were analyzed in this study. Samples were fresh frozen immediately after surgery and stored at -80°C. All specimens analyzed contained more than 40% tumor cells (of the total number of cells counted). The majority of tumors were invasive ductal carcinomas, T1/T2, N0/N1 and histological grade 2 and 3 (Additional file 6). Total RNA was isolated by phenol-chloroform extraction (TRIzol reagent, Invitrogen), the quality and integrity of the total RNA was evaluated on the 2100 Bioanalyzer (Agilent Technologies) and the concentration was measured using a NanoDrop spectrophotometer (NanoDrop Technologies).

### Applied Biosystems expression array analysis

The Applied Biosystems Human Genome Survey Microarray (P/N 4337467) contains 31,700 60-mer oligonucleotide probes representing 27,868 individual human genes. Digoxigenin-UTP labeled cRNA was generated and amplified from 2 μg of total RNA from each sample using Applied Biosystems Chemiluminescent RT-IVT Labeling Kit v 1.0 (P/N 4340472) according to the manufacturer's protocol (P/N 4339629). Array hybridization was performed for 16 hrs at 55 °C. Chemiluminescence detection, image acquisition and analysis were performed using Applied Biosystems Chemiluminescence Detection Kit (P/N 4342142) and Applied Biosystems 1700 Chemiluminescent Microarray Analyzer (P/N 4338036) following the manufacturer's protocol (P/N 4339629). Images were auto-gridded and the chemiluminescent signals were quantified, corrected for background, and finally, spot-and spatially-normalized using the Applied Biosystems 1700 Chemiluminescent Microarray Analyzer software v 1.1 (P/N 4336391). A total of 40 microarrays were used for the analysis: Two process replicates (independent labeling and independent hybridization process) were generated for each of 10 samples, and two technical replicates (the same pool of labeled cRNA and then split into two independent array hybridizations) were generated for the remaining 10 samples. For inter-array normalization, we applied global median normalization across all microarrays to achieve the same median signal intensities for each array.

### Stanford human cDNA microarray analysis

The same RNA samples were also analyzed using Stanford Human cDNA microarrays, which contain 42,000 features representing 24,271 unique cluster IDs (UniGene Build Number 173), manufactured by the Stanford Functional Genomic Facility [39]. Amplification was performed based on the Van Gelder and Eberwine method [40] using the MessageAmp amplification kit (Ambion) and labeling of 3 μg total RNA per sample was carried out as previously described using incorporation of Cy5 for tumor RNA and Cy3 for reference RNA (Stratagene UHR) [41]. Hybridization at 65°C was performed overnight, the hybridized arrays were scanned on an Agilent DNA microarray scanner and images analyzed by GenePix Pro v 4.1. The Limma package (R/Bioconductor) [42] was used to perform within array normalization (print-tip loess normalization) and between array normalization (median of absolute deviation normalization).

### Agilent whole human genome microarray analysis

The Agilent Whole Human Genome Oligo Microarray contains 44,000 60-mer oligonucleotide probes representing 41,000 unique genes and transcripts [43]. Amplification and labeling of 500 ng of total RNA was performed according to the manufacturer's protocol using Cy5 for tumor RNA and Cy3 for the reference RNA (Stratagene UHR). Hybridization was performed for 16 hrs at 50°C and arrays were scanned on an Agilent DNA microarray scanner. Images were analyzed and data were extracted using Agilent Feature Extraction Software A.7.5.1. Lowess normalization was performed for within array normalization between the two channels and a linear scaling (geometric mean of each channel signal is set to a value of 1000) was performed for between array normalization.

### Cross-mapping between microarray platforms

All target transcripts of Applied Biosystems Human Genome Survey Microarray were identified by mapping the 60-mer probe sequences to all transcript sequences from both Celera and public databases including Celera hCT, RefSeq NMs, GenBank mRNAs, MGCs, dbESTs, GenBank CDS, Ensembl cDNA; all target transcripts represented on the Stanford cDNA microarray and the Agilent Whole Human Genome Oligo Microarray were identified by the targeted GenBank accession numbers representing the corresponding probes as specified by each manufacturer. All transcript sequences were then mapped to the Celera-assembled human genome (Build R27) and a transcript-to-gene-clustering was performed so that each transcript could be traced to a gene (removing redundancy) (Xiao C. et al, manuscript in preparation). The common target transcrips between Applied Biosystems Human Genome Survey Microarray and the Stanford Human cDNA microarray (17,732 genes) and between the Applied Biosystems Human Genome Survey Microarray and the Agilent Whole Human Genome Oligo Microarray (22,507 genes) respectively, were identified based on their common cluster membership, respectively. Intersect genes (16,611 genes; see Additional file 3) of these two gene lists were used as the common genes among all three platforms.

### TaqMan^® ^assay-based real-time PCR

mRNA expression of 85 target genes and 4 endogenous control genes was measured in each of the 20 biopsy specimens by real-time PCR using TaqMan^® ^Gene Expression Assays and the ABI PRISM^® ^7900 HT Sequence Detection System (Applied Biosystems, Foster City, CA). Four replicates were run for each gene for each sample in a 384-well format plate. The probes contain a 6-carboxy-fluorescein phosphoramidite (FAM™ dye) label at the 5' end of the gene and a minor groove binder and non-fluorescent quencher at the 3' end and are designed to hybridize across exon junctions. ~ 4 μg of total RNA from each tumor sample was used to generate cDNA using the ABI High Capacity cDNA Archiving Kit (Applied Biosystems, Foster City, CA) and the real-time PCR reactions were carried out following the manufacturer's protocol. TaqMan^® ^Gene Expression Assay IDs are listed in Additional file 6. Among the four measured endogenous control genes (RPS18/PPIA (Alias: cyclophilin A)/GAPDH/PGK1) we chose PPIA for normalization across different genes based on the fact that this gene showed the most relatively constant expression in different breast carcinomas (see Additional file 7).

### Statistical analysis

Statistical analyses were performed with the software packages MATLAB^® ^(Mathworks, Natick, MA), R/Bioconductor [42] GeneSpring (Agilent Technologies, CA) and Spotfire Functional Genomic (Spotfire, Göteborg, Sweden).

#### Centroid correlation analysis

An "intrinsic" gene list consisting of 534 genes represented by 552 clones, was previously selected based on their low variation in expression in successive samples from the same patient's tumor and at the same time, high degree of variation among tumors from different patients [10]. These intrinsic genes have been used to define five subtypes of breast tumors and their core expression centroids (i.e., average expression profile of the 534 intrinsic genes) in a data set of 122 breast tissue samples, most of which were locally advanced breast tumors. 526 of the intrinsic genes were mapped to the corresponding genes represented on the Applied Biosystems Human Genome Survey Microarray and 510 were mapped among all three microarray platforms used in this study. Using these mapped genes, we computed the Pearson's correlation coefficient of each sample from this study to each of the five centroids and assigned each sample to the subtype with which it showed the highest correlation.

#### Hierarchical clustering

Average-linkage hierarchical clustering analysis and visualization was performed using the Cluster and TreeViev programs [44]. When multiple platform data were analyzed together, each data set was first normalized between arrays and between genes independently, and then combined for clustering analysis. For the single-color Applied Biosystems microarray platform, gene expression signals were first normalized between arrays to the same median expression level in log2 space, and then normalized by median expression level or by z-score transformation across all samples for each gene. For the two-color array systems (Stanford cDNA microarrays and Agilent oligo arrays), normalization within and between arrays were performed using the log2 ratio of the sample vs. the reference as described earlier, and then normalized by median expression ratio or by z-score transformation across all samples for each gene. For TaqMan^® ^assay-based real-time PCR, -ΔCt = Ct_endogenous control – Ct_gene was calculated as an equivalent of normalized relative gene expression level, and then z-score-transformed across all samples for each gene. The z-score was determined as number of standard deviations of the level of expression of each gene in each sample away from the mean level of expression of that gene across all the samples within the data set of the given platform.

#### PAM

Class prediction was performed by using prediction analysis of microarrays (PAM), a statistical package [45] that applies nearest shrunken centroid analysis and cross-validation to determine a minimal set of predictor genes that achieve optimal prediction accuracy for sample classification [46].

#### Differential expression analysis

Welch-ANOVA analysis coupled with Benjamini and Hochberg False Discovery Rate multiple testing corrections were performed using GeneSpring software package to identify the most diferentially expressed genes between the luminal A and basal-like subtypes.

#### PANTHER™ protein classification system analysis

Similar to Gene Ontology™ (GO), PANTHER™ (Protein ANalysis THrough Evolutionary Reationships) Protein Classification System (Applied Biosystems, Foster City, CA) [47] classifies proteins in families/sub-families, molecular functions, biological processes and biological pathways. Compared to GO, the PANTHER™ Protein Classification System provides a more simplified ontology (vocabulary) of protein function and classifies 25% more proteins than GO [48]. Protein classification over-represented by "signature" genes of the luminal A and the basal subtype were identified and the statistical significance of the over-representation was quantified by a random overlapping *p *value using the binomial test with all the genes represented by the Applied Biosystems Human Genome Survey Microarray as the reference list [49].

#### Pathway analysis

Pathway analysis was performed using PathArt™ (Jubilant Biosys Ltd., Mahalakshmipuram, Bangalore). PathArt is a curated database of biomolecular interactions with more than 1400 regulatory and signaling pathways. Compared to a few publicly available pathway databases (i.e. GenMapp [50], KEGG [51]), which tend to be heavily enriched in metabolic pathways, the PathArt database emphasizes more on disease specific networks and regulatory and signaling pathways. The statistical significance of the over-representation of the given pathway within each "signature" gene list was quantified as a similarity p-value (likelihood of a random overlapping) using script SG3b-1 (BioScripts 2.1, GeneSpring software) based on Fisher's Exact Test.

#### Profile correlation between microarray and TaqMan^® ^assay-based real-time PCR data

Data sets from each microarray platform and TaqMan^® ^Gene Expression assays were normalized sample-wise and gene-wise (z-score transformation) as described above. Pearson's correlation coefficient (R) was calculated between the expression profile for each of the 85 validation target genes across the 20 tumor samples determined by each microarray platform and the expression profile determined by TaqMan^® ^Gene Expression assays.

#### GEO accession

The data from three microarray platforms and the TaqMan gene expression assays have been deposited in Gene Expression Omnibus (GEO 3155) [52].

## Competing interests

YW and RRS are employees of Applied Biosystems, CX is an employee of Celera Genomics.

## Authors' contributions

TS and YW conceived, designed the study, performed the experiments, analyzed data and wrote the article. CX performed cross-platform gene mapping. HJ performed experiments. BN contributed with clinical samples and discussions. RRS and ALBD contributed to conception and design of the study and revising and writing of the article. All authors read and approved the manuscript.

## Supplementary Material

Additional File 1 **The 1210 signature genes represented by 1244 probes that were most differentially expressed between luminal A and basal-like tumors by ANOVA analysis**. For each gene, the Applied Biosystems Probe ID, P-value, Fold Change, Gene_Symbol, Gene_Description, GenBank Accession and LocusLink_ID are listed. Click here for file

Additional File 2 **Descriptive pathway diagrams for the luminal A and basal-like subtypes**. (A) ER signaling pathway is over-represented by the luminal A signature genes. (B) p21-mediated signaling pathway is over-represented by the basal-like signature genes. Click here for file

Additional File 3 **The 16611 common genes mapped among the three microarray platforms used in this study**. This file contains a gene list of 16611 genes with their corresponding Applied Biosystem Human Genome Survey Microarray Probe IDs, Agilent Human Whole Genome Oligo Microarray Probe IDs and Stanford Human 42 k cDNA array SUIDs. Click here for file

Additional File 4 **54-gene set for discrimination between luminal A and basal-like subtypes**. This file contains the minimal set of 54 genes that best discriminated luminal A and basal-like tumors by PAM analysis. Click here for file

Additional File 5 **TaqMan^® ^Gene Expression assays used in this study**. This file contains a gene list of 85 genes with their corresponding TaqMan^® ^Gene Expression Assay IDs, Applied Biosystem Human Genome Survey Microarray Probe IDs, Agilent Human Whole Genome Oligo Microarray Probe IDs, and Stanford Human 42 K cDNA Array SUIDs. Click here for file

Additional File 6 **Tumor characteristics of the 20 samples analyzed in this study**. Tumor size (cm); molecular subtype (uc = unclassified); tumor category (tcat) given as T size; nodal status (ncat); histological grade; tumor cell content. Click here for file

Additional File 7 **Expression profiles of four tested endogenous control genes in various breast cancer tissues**. PPIA (Cyclophilin A) was chosen as the endogenous control as this gene showed the most relatively constant expression levels (smallest standard deviation and variance) across different breast carcinomas. Click here for file
