# Supplementary material for: Distinct molecular mechanisms underlying clinically relevant subtypes of breast cancer: gene expression analyses across three different platforms
Source: BMC Genomics. 2006 May 26;7:127. doi: 10.1186/1471-2164-7-127 (PMC1489944; doi:10.1186/1471-2164-7-127)
Supplement: Additional File 6 — Tumor characteristics of the 20 samples analyzed in this study. Tumor size (cm); molecular subtype (uc = unclassified); tumor category (tcat) given as T size; nodal status (ncat); histological grade; tumor cell content. [file 1471-2164-7-127-S6.doc]

| **sample ID** | **molecular tumor subtype (1=Lum A, 2= Lum B, 3=ERBB2+, 4= Basal-like, 5= Normal-like)** | **tumor size** | **tcat(1=T1, 2=T2, 3=T3, 4=T4, 7=not classifiable)** | **ncat (N0=0, N1=1, not classifiable =7))** | **grade** | **% tumor cells (of no. of cells counted)** |
| --- | --- | --- | --- | --- | --- | --- |
| 20 | 5 | 1 | 1 | 0 | 2 | 40 |
| 31 | 4 |  | 1 | 0 | 3 | 80 |
| 42 | 4 | 1.4 | 1 | 0 | 3 | 80 |
| 53 | 3 | 4 | 2 | 0 | 2 | 100 |
| 65 | 1 |  | 1 | 1 | 1 | 90 |
| 67 | 4 | 2.7 | 2 | 1 | 3 | 95 |
| 79 | 3 | 4 | 4 | 1 | 3 | 95 |
| 85 | 1 | 4.5 | 2 | 1 | 3 | 90 |
| 88 | 1 | 3.5 | 2 | 1 | 3 | 90 |
| 91 | Uc |  | 2 | 0 | 3 | 100 |
| 101 | 1 | 2.4 | 2 | 0 | 2 | 90 |
| 122 | 1 | 1.5 | 1 | 0 | 2 | 80 |
| 132 | Uc | 3 | 2 | 1 | 2 | 90 |
| 146 | 3 | 3.5 | 2 | 1 | 2 | 65 |
| 148 | 2 | 3.2 | 2 | 0 | 3 | 95 |
| 185 | 4 | 2.1 | 2 | 1 | 3 | 100 |
| 263 | 1 |  | 7 | 1 | 2 | 65 |
| 267 | 4 |  | 7 | 1 | 3 | 95 |
| 632 | 1 | 6 | 3 | 1 | 2 | 70 |
| 709 | 4 | 2.5 | 2 | 0 | 3 | 80 |
